# Molecular identification of human enteroviruses associated with aseptic meningitis in Yunnan province, Southwest China

**DOI:** 10.1186/s40064-016-3194-1

**Published:** 2016-09-08

**Authors:** Yanju Zhu, Xi Zhou, Jiansheng Liu, Longhui Xia, Yue Pan, Junying Chen, Na Luo, Jianzhong Yin, Shaohui Ma

**Affiliations:** 1 Institute of Medical Biology, Chinese Academy of Medical Sciences, and Peking Union Medical College (CAMS and PUMC), 935 Jiao Ling Road, Kunming, 650118 Yunnan Province People’s Republic of China; 2 Yunnan Key Laboratory of Vaccine Research Development on Severe Infectious Disease, Kunming, 650118 People’s Republic of China; 3 Xixi Hospital of Hangzhou, Hangzhou, 310023 People’s Republic of China; 4 School of Public Health, Kunming Medical University, 1168 West Chun Rong Road, Yuhua Avenue, Chenggong District, Kunming, 650500 Yunnan People’s Republic of China

**Keywords:** Genotyping, Phylogenetic analysis, Echovirus 30, Coxsackievirus B5, Echovirus 9

## Abstract

*Human enteroviruses* (EVs) are the major causative agents of aseptic meningitis. In this study, a total of 524 children were admitted to the children Kunming hospital (continental China) for aseptic meningitis manifestations in 2009 and 2010. An EV infection was diagnosed in 85/524 children (16.2 %) and the viruses detected were assigned to 16 serotypes. Most serotypes belonged to the enterovirus B species. Echovirus 9 was predominant (24.7 %), followed by coxsackievirus B5 (23.5 %) and then echovirus 30 (16.5 %). Echovirus 9 was firstly identified as the predominant serotype in sporadic aseptic meningitis which occurred in Yunnan, Southwest China. This work indicates the need to perform large-scale surveillance to gain a better insight into the epidemiology of enteroviruses associated with aseptic meningitis in China.

## Background

*Human enteroviruses* (EVs) belong to the family *Picornaviridae*, and they are classified into four taxonomic species: EV-A, B, C, and D (Lo et al. 2010). EVs comprise more than 100 distinct serotypes. Although most EV infections are asymptomatic or mild, they can also result in diseases of the central nervous system (CNS), including aseptic meningitis, encephalitis, acute flaccid paralysis, paralytic myelitis, and cerebellar ataxia (Othman et al. 2016). EVs are the major causative agents of aseptic meningitis in many countries (Dalwai et al. 2010; Fowlkes et al. 2008; Jain et al. 2014; Zhao et al. 2005). Coxsackievirus (CV) A9, A10, B3, and B5; echovirus (E) 4, 5, 9, 11, 19, and 30; and EV-A71, EV-75, 76, and 89 have often been reported in sporadic meningitis cases and epidemics (Dalwai et al. 2009; Kumar et al. 2011; Lewthwaite et al. 2010; Lin et al. 2003; Sapkal et al. 2009).

The EV genome is a positive single-stranded RNA molecule of approximately 7500 nt, comprising a single open reading frame (ORF) flanked by 5′- and 3′-untranslated regions (UTRs). The ORF is divided into three subregions, P1, P2, and P3. The P1 region encodes four structural proteins (VP4, VP2, VP3, and VP1) in a sequential order from the 5′ non-coding region. The non-structural proteins are encoded in the P2 (2A, 2B, and 2C) and P3 (3A, 3B, 3C, and 3D polymerase) regions. The VP1 capsid protein is the most external and immunodominant of the picornavirus capsid proteins and contains neutralization epitopes. VP1 sequences correlate well with antigenic typing by neutralization tests, and can be used for EV identification and molecular epidemiology (Oberste et al. 1999).

A number of EV types such as E-30, E-6, CV-A9, CV-B3, and CV-B5 were involved in outbreaks of aseptic meningitis in China (Zhao et al. 2005; Chen et al. 2013; Cui et al. 2010; Mao et al. 2010; Tao et al. 2012; Wang et al. 2006). In this study, we describe the results obtained following molecular typing of EVs detected in clinical samples obtained in patients with sporadic aseptic meningitis in Yunnan, China from 1 May 2009 to 11 May 2010, with an emphasis on exploring their epidemiological and genetic characteristics.

## Methods

### Sample collection

From 1 May 2009 to 11 May 2010, stool and cerebrospinal fluid (CSF) specimens were collected in children admitted with meningitis symptoms to a pediatric hospital in Kunming (China), and were sent to our laboratory for analysis within 24 h. This clinical symptoms and signs of aseptic meningitis were as follows: fever, vomiting, headache, convulsion, lethargy, neck stiffness. Stool and CSF specimens were maintained at about 4 °C during sample transport, and stored at −70 °C.

Patients’ demographical data, clinical symptoms, and major complications were collected retrospectively from medical history. This work was approved by the Institutional Review Boards of the Institute of Medical Biology, Chinese Academy of Medical Sciences and Peking Union Medical College. All participants gave written informed consent. The protocol was in accordance with the Helsinki Declaration.

### Virus RNA extraction, RT-PCR and sequencing

To identify the enterovirus serotypes associated with aseptic meningitis, a nested-PCR was performed on stool specimens from the patients. Briefly, 1 g of stool specimen was suspended in 5 mL of phosphate buffered saline (PBS), and the suspensions were centrifuged at 4 °C, 3000×*g* for 30 min. The supernatant was transferred into a new tube and stored at −80 °C. Viral RNA was extracted from the samples by a QIAamp viral RNA Mini Kit (QIAGEN, Valencia, CA, USA) according to the manufacturer’s recommended procedure.

Reverse transcription polymerase chain reaction (RT-PCR) and PCR were done using Primescript one-step RT-PCR Kit Ver. 2 and 2× Taq PCR Master Mix (TakaRa, Dalian, China) according to the manufacturer’s instructions. Nested primers were used for the detection of enterovirus based on the standard protocol (Leitch et al. 2009). The 25-µl reaction system was composed of 1 µl RT-PCR mix, 12.5 µl 2× Reaction Buffer, 20 pmol of A-OS and A-OAS primer for EV-A or B-OS and B-OAS for EV-B, and 8 µl RNA. The amplification conditions were as follows: reverse-transcripting at 50 °C for 30 min, pre-denaturing stage at 94 °C for 2 min, denaturing stage at 94 °C for 30 s, annealing stage at 52 °C for 30 s and elongating stage at 72 °C for 1 min; 30 cycles were performed. Subsequently, the second-round PCR setup was performed with the former PCR products and the A-IS and A-IAS primers for EV-A or B-IS and B-IAS for EV-B, as described above. The PCR products were subjected to electrophoresis on a 1 % agarose gel to identify positive samples with the predicted size of amplicons. All positive products were sequenced directly by using an ABI 3730XL automatic sequencer (Applied Biosystems, Foster City, CA, USA).

### Enterovirus typing and phylogenetic analysis

The partial or complete VP1 sequences obtained were compared with sequences available in GenBank using basic local alignment (BLAST, http://www.ncbi.nlm.nih.gov/BLAST). The viral sequences that displayed more than 75 % nucleotide similarity (85 % amino acid identity) were considered to be of the same serotype. Phylogenetic trees were constructed by Mega 6.06 using neighbor-joining after estimation of genetic distance using the Kimura two-parameter method. A bootstrapping test was performed with 1000 replicates.

### Statistical analysis

Statistical analysis was performed using SPSS (Statistical Package for the Social Sciences) 12.0 software (Chicago, IL, USA). Two-tailed *P* values <0.05 were considered significant.

### Nucleotide sequence accession numbers

The sequences described in this study have been deposited in the GenBank database, under accession numbers KU665301–KU665345.

## Results

### Cases and epidemiology

A total of 524 cases were reported during the study period, of which 85 (16.2 %) were positive for EVs. Patients’ age ranged between 3 months 13 days to 14 years old. The gender ratio was 1.34:1, with 300 male and 224 female cases, indicating a greater incidence of EV associated aseptic meningitis in male children.

The annual distribution of clinical features and laboratory findings of children with aseptic meningitis associated with EV and other viruses were indicated in Table 1. There were no significant differences between EV-associated aseptic meningitis (EVAM) and other-virus-associated aseptic meningitis (OVAM) groups in demographic features. The data showed that the frequency of headache (*P* < 0.01) was significantly higher in EVAM than in OVAM.

**Table 1:** Comparative demographic and clinical features of aseptic meningitis patients associated with enterovirus and other virus in the present study

Variable	EVAM (n = 85)	OVAM (n = 439)	*P* value
Demographics			
Age (years, mean ± SD)	5.6 ± 3.0	5.3 ± 3.1	NS
Sex [no. (%)]			
Male	50 (58.8)	250 (56.9)	NS
Female	35 (41.2)	189 (43.1)	
Clinical symptoms [no. (%)]			
Fever	67 (78.8.)	388 (88.4)	0.017
Headache	60 (70.6)	241 (54.9)	0.007
Neck stiffness	46 (54.1)	254 (57.9)	NS
Vomitting	62 (72.9)	284 (64.7)	NS
Convulsion	5 (5.8)	73 (16.6)	0.011
Lethargy	9 (10.6)	73 (16.6)	NS
Diarrhea	3 (3.5)	9 (2.0)	NS
Laboratory findings [no. (%)]			
EEG abnormal	50 (58.8)	261 (59.5)	NS
Skull CT abnormal	24 (28.2)	102 (23.2)	NS
CSF pleocytosis	36 (42.4)	199 (45.3)	NS
Elevated CSF protein	25 (29.4)	140 (31.9)	NS

*NS* not significant, *EVAM* enterovirus-associated aseptic meningitis, *OVAM* other-virus-associated aseptic meningitis

### Enterovirus typing

VP1 amplification was performed on all 524 samples and 85 samples (16.2 %) had positive results. Further VP1 sequencing and molecular typing revealed all sequences obtained were assigned to 16 different EV serotypes (Table 2). E-9 was the most frequently detected serotype (21/85 cases, 24.7 %), followed by CV-B5 (20/85, 23.5 %) and E-30 (14/85, 6.5 %). The low rate of molecular typing of clinical specimens positive for EVs may relate to low viral load of the samples or other virus infection.

**Table 2:** Enterovirus types identified from 524 samples in Yunnan province, Southwest China

Enterovirus serotype	Number detected in	Percentage of total (%)
Coxsackievirus A16	3	3.5
Coxsackievirus A9	3	3.5
Coxsackievirus B2	6	7.1
Coxsackievirus B3	2	2.4
Coxsackievirus B4	2	2.4
Coxsackievirus B5	20	23.5
Echovirus 4	1	1.2
Echovirus 6	4	4.7
Echovirus 9	21	24.7
Echovirus 15	1	1.2
Echovirus 16	4	4.7
Echovirus 18	1	1.2
Echovirus 21	1	1.2
Echovirus 30	14	16.5
Echovirus 33	1	1.2
Echovirus 12	1	1.2
Total	85	–

### Phylogenetic analysis and homologous comparison

Dendrograms were constructed based on the VP1 sequences from identified E-9, CV-B5, and E-30, respectively.

**Fig. 1:**
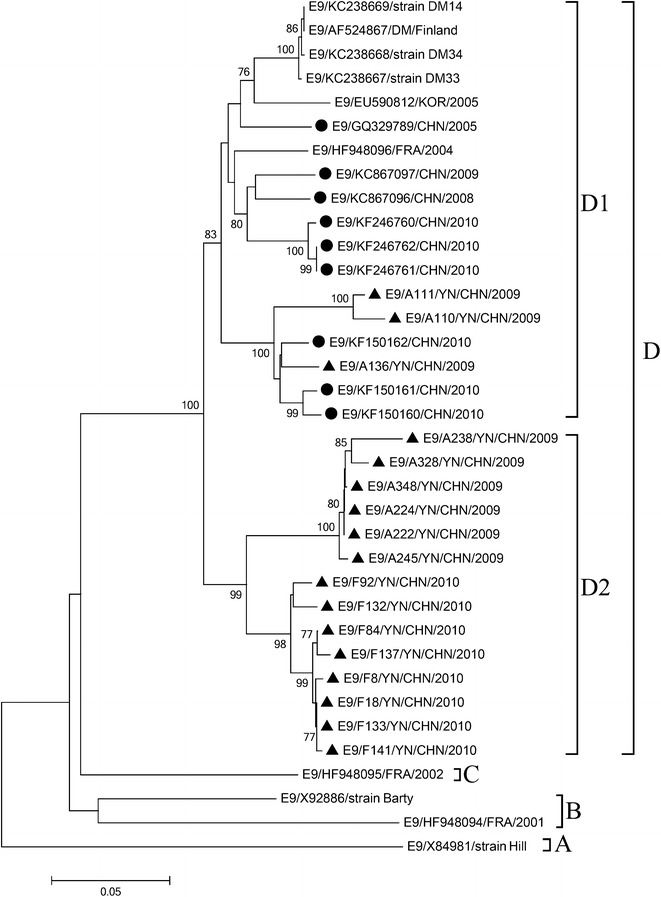
Phylogenetic trees based on the VP1 gene sequences (906 bp) of E-9 generated by the neighbor-joining algorithm implemented in MEGA (version 6.1) using the Kimura two-parameter substitution model and 1000 bootstrap pseudo-replicates. Only strong bootstrap values (>75 %) are shown. *Filled triangle* strains isolated in this investigation. *Filled circle* China

All E-9 in this study were classified into two distinct genetic lineages (Fig. 1), implying that the E-9 isolated in this sporadic case belonged to two different transmission chains. The nucleotide and amino acid sequence similarities of the VP1 gene between the two lineages were 87.4–91.5 and 90.7–96.7 %, respectively. The nucleotide and amino acid sequence similarities between the E-9 isolates and other Chinese E-9 strains within the VP1 gene sequences were 87.5–93.5 and 95.7–98.0 %, respectively. Most of the E-9 isolates were independently clustered into one of the two lineages, whose nucleotide and amino acid similarities ranged from 97.7–100 and 94.0–100 %, respectively.

**Fig. 2:**
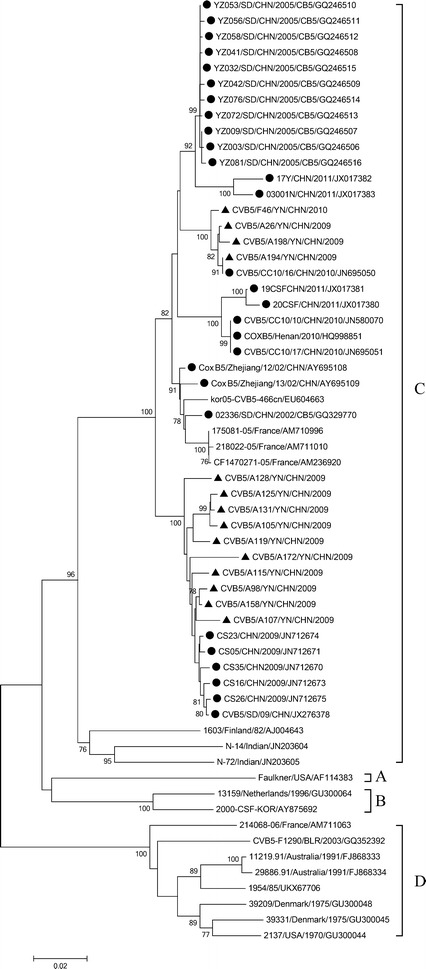
Phylogenetic trees based on the VP1 coding sequences (849 bp) of CV-B5 generated by the neighbor-joining algorithm implemented in MEGA (version 6.1) using the Kimura two-parameter substitution model and 1000 bootstrap pseudo-replicates. Only strong bootstrap values (>75 %) are shown. *Filled triangle* strains isolated in this investigation. *Filled circle* China

CV-B5 was segregated into four lineage groups A–D (Fig. 2), which were assigned to previously reported lineages (Chen et al. 2013). All Chinese strains belonged to C group, which further formed into two distinct lineages. CV-B5 isolates in the study were clustered into two lineages, respectively. The nucleotide sequence and amino acid similarities between the two clusters within the VP1 gene were 92.1–94.2 and 92.4–94.5 %, respectively. The nucleotide and amino acid similarities between the CV-B5 isolates and other Chinese CV-B5 strains within the VP1 gene were 91.1–97.3 and 98.2–99.8 %, respectively.

**Fig. 3:**
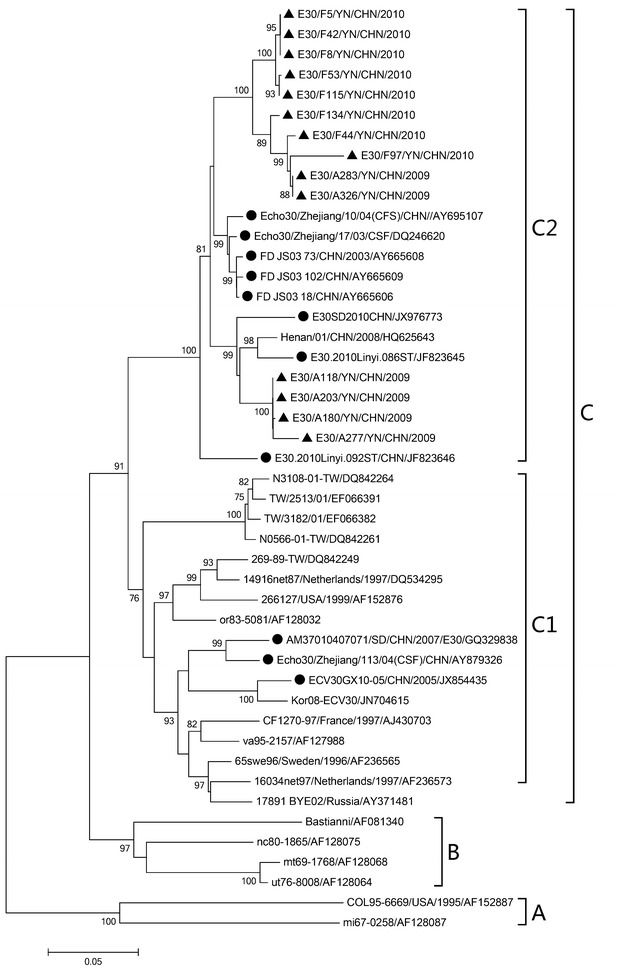
Phylogenetic trees based on the VP1 coding sequences (876 bp) of E-30 generated by the neighbor-joining algorithm implemented in MEGA (version 6.1) using the Kimura two-parameter substitution model and 1000 bootstrap pseudo-replicates. Only strong bootstrap values (>75 %) are shown. *Filled triangle* strains isolated in this investigation. *Filled circle* China

The E-30 sequences determined in the study clustered within 3 virus lineages, which were assigned to previously reported genogroups, A-C (Fig. 3) (Zhao et al. 2005; dos Santos et al. 2011). All Chinese strains belonged to group C, which further formed two distinct clusters. E-30 isolates in the study were clustered into one of them. The nucleotide and amino acid similarities between the E-30 isolates in the study and other Chinese E-30 strains within the VP1 gene were 84.1–94.9 and 95.2–99.3 %, respectively.

## Discussion

More than 100 viruses can cause aseptic meningitis (Johnson 1996). Herpes simplex virus (HSV), Varicella zoster virus (VZV) and EVs are the most common viral causes (Hosoya et al. 1998, 2002). Though, the surveillance system of aseptic meningitis is very limited, several related studies conducted in China have shown that EVs were the predominant pathogen (Xie et al. 2015; Zhang et al. 2013). Previous studies show that EVs were responsible for about 16 % of aseptic meningitis cases in pediatric and adult populations (Archimbaud et al. 2009; Dos Santos et al. 2006), which is consistent with our report. EVs associated with aseptic meningitis outbreaks mainly focus on E-30, E-6, CV-A9, CV-B3, and CV-B5 (Zhao et al. 2005; Chen et al. 2013; Cui et al. 2010; Mao et al. 2010; Tao et al. 2012; Wang et al. 2006). Those serotypes were also included in the most commonly reported serotypes in EVs surveillance from the USA (Abedi et al. 2015). In addition, it was unclear why male had more chances to get infected. But, now it is generally believed that they spent more time on playing than female and had many opportunities to be exposed to the viruses.

In the past decades, Hand, foot and mouth disease (HFMD) caused by EVs has become more predominant in the Asia–Pacific region. Especially after 2008, HFMD has become a pressing issue for public health in China. EV associated HFMD accompanying aseptic meningitis have been reported, such as EV-A71, CV-A16, CV-A6, CV-A10, CV-A4, and E-9, and some of them have become the main causative agents of aseptic meningitis for epidemics of HFMD (Huang et al. 1999, 2015). Our previous study has shown that EV-A71, CV-A16, and E-9 were the most common serotypes detected for HFMD and other serotypes such as CV-A1, CV-A8, CV-A9, CV-A10, CV-B2, CV-B4, E-1, and E-3 have been identified from 2009 to 2011 (Ma et al. 2015). In this study, CV-A16, CV-A9, CV-B2, CV-B4, E-4, E-6, E-9, E-12, E-15, E-16, E-18, E-21, and E-33 were detected. E-9, CV-B5, and E-30 were the most common serotypes detected for aseptic meningitis from 2009 to 2010. As a rule, the main serotypes of causative agents for HFMD and aseptic meningitis are different (EV-A for HFMD, while EV-B for aseptic meningitis), but overlaps of the causative agents exist (Tao et al. 2014). Thus, the main serotypes of causative agents for HFMD may overlap with that of aseptic meningitis. In addition, the identification rate of EV types is very low in the present study by compared with our previous study. This is because EVs are causative agents of HFMD, and EVs are only the most common causative viruses of aseptic meningitis. Other viruses can also cause aseptic meningitis. So we speculate it may be related to other virus infection except the viral load of the samples.

The average nucleotide divergence of VP1 was <13.36 % (9–17 %) and 4.08 % (1–10 %) and may be considered the same inter- and intra-subgenotypes, respectively (Chan et al. 2010). Although E-30 and E-9 strains detected in this study belonged to a single genogroup within each type, they clustered within distinct subgenogroups. Accordingly, the contemporaneous Chinese E-30 and E-9 might have evolved along two different pathways (C1 and C2, D1 and D2). The different clusters may be the result of genetic drift that commonly occurs among the *Picornaviridae*. The different clusters for these EVs are formed by 2009 and 2010 isolates. For CV-B5, there was only a single 2010 isolate.

E-9 was regularly detected in other countries (El et al. 2012; Wolfaardt et al. 2014). In China, E-9 was regarded previously as a rare serotype. In the study, E-9 was predominant, followed by CV-B5, E-30 and CV-B2 in China. However, in Spain, the commonest serotype detected was E-30, followed by E-6, E-13, E-11, and E-9 (Trallero et al. 2010) from 1998 to 2007; E-4 was the most frequently identified, followed by E-30, E-9, and E-6 in 2008 (Cabrerizo et al. 2008). Thus many serotypes exist at rather low levels, other serotypes occur sporadically or outbreak and a previously rare serotype may become predominant. E-9 may also have been considered an emerging human pathogen as CV-A6 and CV-A10, which have become the main pathogens of HFMD (Blomqvist et al. 2010; Xu et al. 2015).

## Conclusion

It is very important to continue surveillance of aseptic meningitis agents in children in order to effectively develop the related vaccines, particularly E-30, which was the most commonly reported serotype (Trallero et al. 2010; Holmes et al. 2016). At present, although EV-A71 and CV-A16 vaccines have been developed, the development of a broadly protective multivalent vaccine against aseptic meningitis or HFMD should include other predominant serotypes of EVs surveyed by years.

## Abbreviations

EVs: human enteroviruses
CNS: central nervous system
CV: coxsackievirus
E: echovirus
CSF: cerebrospinal fluid
EVAM: enterovirus-associated aseptic meningitis
OVAM: other-virus-associated aseptic meningitis
HFMD: hand, foot and mouth disease
